# Design and implementation of an adjustable Micro PDLC Driver for smart buildings

**DOI:** 10.1016/j.ohx.2025.e00648

**Published:** 2025-04-15

**Authors:** Kun-Che Ho, Rui-Feng Xu, Cheng-Xun Wu, Jia-Zheng Liao

**Affiliations:** Department of Automation Engineering, National Formosa University, Yulin, Taiwan

**Keywords:** Green building, Energy saving, PDLC, Micro driver

## Abstract

Polymer Dispersed Liquid Crystal (PDLC) glass, with its controllable light transmittance enabling shading and energy savings, is widely used in green and smart buildings as a key technology for smart windows and privacy glass. However, traditional PDLC drivers are bulky, consume high energy, and offer limited functionality, restricting their application in multi-panel glass control and space-constrained scenarios. This study proposes a low-power, adjustable mini driver that utilizes Pulse Width Modulation (PWM) signals and a full-bridge inverter architecture to generate AC square waves. By integrating simple analog and digital circuit designs, digital resistors, and specialized adjustable power modules, remote voltage and frequency modulation control is achieved, enabling efficient and flexible PDLC driver development. Compared to traditional transformer designs, the developed driver not only offers the advantages of miniaturization and high efficiency but also can flexibly adapt to diverse application scenarios such as office privacy glass, smart buildings, and multi-zone linkage control. This research provides an effective solution for the widespread application of PDLC technology and the advancement of smart buildings. Finally, the functionality of the proposed design is verified through hardware circuit implementation and experimental validation.

## Graphical abstract

**Figure:**
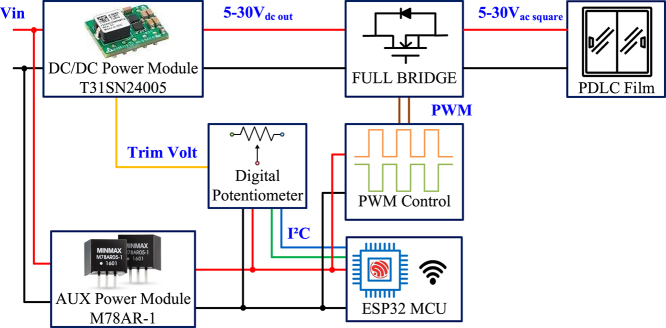

## Specifications table

**Table:** 

**Hardware name**	Micro PDLC Driver with IoT Functionality

**Subject area**	• Power electronics • Electrical engineering • Engineering and material science • Smart Building Technology

**Hardware type**	• Power electronics • Electrical engineering

**Closest commercial analog**	The closest commercial equivalent product is an AC–AC converter. The proposed design in this study uses a DC input, allowing compatibility with various chargers available on the market. With its open modular architecture and IoT functionality, it offers advantages that make it a suitable replacement for such commercial products in customized designs.

**Open source license**	CC BY 4.0

**Cost of hardware**	US$ 64

**Source file repository**	Source files repository (OSF) write the DOI URL here. http://dx.doi.org/10.17605/OSF.IO/WGK7Q

## 1 Hardware in context

Polymer Dispersed Liquid Crystal (PDLC) glass is a modern advanced material composed of liquid crystal droplets uniformly dispersed in a polymer matrix, forming a composite film with anisotropic characteristics [1], [2], [3]. Due to its ability to switch rapidly between transparent and opaque states under an applied or removed voltage, PDLC has been widely utilized in smart windows, display technologies, and green buildings. In smart windows, it enables adjustable light control and privacy, enhancing energy efficiency. In display applications, PDLC provides high contrast and fast response times. Additionally, its use in green buildings helps reduce energy consumption by optimizing natural lighting and thermal insulation [4], [5], [6], [7], [8], [9], [10]. Its fast response speed makes it an ideal choice for applications in energy-efficient buildings and privacy protection. The core working principle of PDLC involves applying appropriate voltage to alter the alignment of liquid crystal molecules, thereby regulating light transmittance [11], [12], [13], [14], [15]. Additionally, as PDLC fabricated with different processes exhibits variations in optical and electrical properties, material selection and optimization must be tailored to specific application scenarios [16], [17], [18], [19]. Although PDLC glass can be driven by direct current (DC), DC driving leads to rapid material aging [14]. Furthermore, Ref. [20] indicates that applying a DC voltage to PDLC films induces a memory state phenomenon, preventing liquid crystal molecules from fully returning to their original random alignment. Over time, this effect accumulates, ultimately reducing the lifespan of the PDLC film. Refs. [21], [22] report that DC voltage application on PDLC films leads to conductive effects and interfacial charge accumulation, weakening the effective electric field and gradually decreasing light transmittance. Furthermore, Ref. [23] highlights two primary limitations of DC-driven PDLC operation: prolonged DC voltage exposure causes oxidation at the electrode interface, and ion fatigue effects contribute to a continuous decline in transmittance. Collectively, these studies suggest that DC voltage driving may result in permanent or unintended molecular alignments, thereby compromising the stability of PDLC transmittance and its long-term reliability. Therefore, using alternating current (AC) to drive PDLC is a more feasible and efficient option.

Currently, most commercial PDLC drivers rely on sine wave voltage provided by mains electricity, with voltage adjustments made through coupling transformers. However, a major drawback of such drivers is their bulky size, primarily due to the large space required by transformers, which makes integration into space-constrained applications challenging. Moreover, traditional drivers have fixed, non-adjustable frequencies, limiting the ability to optimize power consumption and performance based on application requirements. This is particularly disadvantageous for long-term operation in smart window or privacy glass systems. Additionally, literature [18] indicates that AC square waves are as effective as sine waves for driving PDLC while being easier to implement and control, making them especially suitable for miniature and flexible driver systems [24], [25], [26].

To address these issues, this study presents a novel PDLC driver designed to provide a compact, cost-effective, and efficient solution. The driver uses two timers to generate complementary PWM signals, which are processed through a full-bridge circuit to convert DC into AC square waves, enabling stable control of liquid crystal alignment and achieving more efficient light transmission regulation [24], [25], [26]. The digital resistor of the driver is controlled by a microcontroller (MCU), allowing precise adjustment of the output voltage range (5 V to 30 V), which corresponds to the effective operating voltage range of PDLC and satisfies the control requirements for multiple PDLC panels. Additionally, the driver reduces power consumption through optimized frequency control, making it particularly suitable for long-duration applications such as smart windows and privacy glass. This design not only significantly reduces size but also extends system lifespan and minimizes energy consumption, providing an efficient and flexible solution for PDLC driver technology.

## 2 Hardware description

### 2.1 Architecture of the micro PDLC driver

**Fig. 1:**
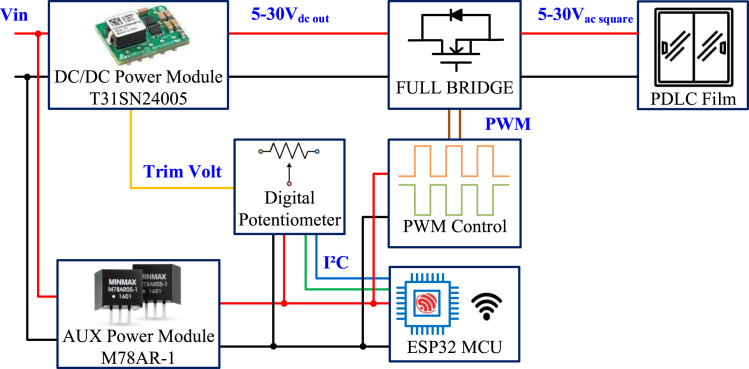
Micro PDLC driver block diagram.

The system architecture of the micro PDLC driver is illustrated in Fig. 1. The overall design is divided into two major parts: the power stage and the control stage. The power stage employs Delta Electronics’ ultra-wide input/output power module T31SN24005 as the primary power supply, combined with Texas Instruments’ dual-channel gate driver UC3707 to generate stable AC square wave outputs, meeting the driving requirements of PDLC glass. The control stage adopts a master–slave architecture based on 555 timers to generate complementary PWM control signals and uses the digital potentiometer AD5245 for precise output voltage regulation. The AD5245 communicates with the control module via the I2C protocol to adjust the output voltage of the T31SN24005 power module, enabling accurate control of the square wave amplitude. Additionally, the system integrates the ESP32 chip as the I2C communication master device to handle remote control requirements, providing convenience for flexible operation during experiments. This architecture combines an efficient power stage design with a flexible digital control strategy, significantly enhancing the efficiency and stability of PDLC glass driving. Moreover, it incorporates remote control functionality, offering a reliable technical solution for applications such as smart windows and privacy glass.

### 2.2 Hardware circuit of the PDLC driver

**Fig. 2:**
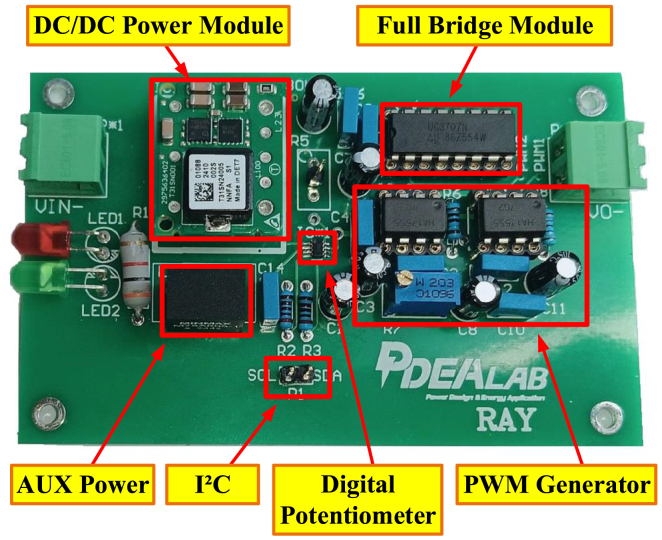
Hardware circuit of the micro PDLC driver.

**Fig. 3:**
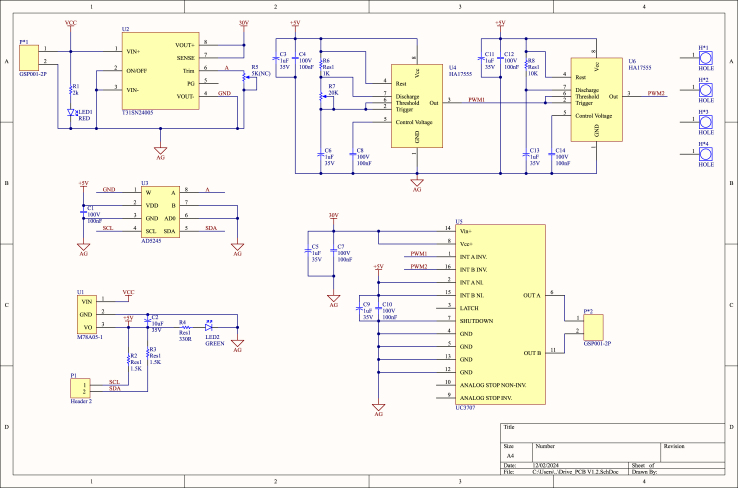
Schematic of the micro PDLC driver.

The hardware circuit and schematic of the developed micro PDLC driver are shown in Fig. 2, Fig. 3, incorporating the previously mentioned subsystem modules. Through modular design, the system not only effectively reduces maintenance costs but also increases circuit integration density, further minimizing the size of the driver. Compared to conventional commercial PDLC drivers, the proposed driver offers significant advantages, including a smaller size, simpler architecture, and lower cost. This innovative solution provides an efficient and economical approach for driving PDLC glass.

### 2.3 Design of the voltage output and amplitude adjustment module

A stable and adjustable output voltage is a critical requirement in designing PDLC drivers, as different light transmittance levels require corresponding voltages to achieve optimal performance. To meet this need, the T31SN24005 DC–DC converter was selected as the primary power module for the system. This converter offers high efficiency, a wide input voltage range (9 V to 53 V), compatibility with various industrial power specifications (e.g., 12 V, 24 V, 48 V), and an ultra-wide adjustable output voltage range (5 V to 30 V). It also supports a maximum output current of 4.5 A, making it suitable for various PDLC panel sizes and transmittance settings, thereby addressing the diverse requirements of PDLC applications. Additionally, the converter features built-in short-circuit and over-voltage protection, significantly enhancing system stability and reliability.

By utilizing the T31SN24005, the proposed driver achieves precise voltage control while maintaining an efficient and compact design, providing a stable and high-performance solution for smart glass applications. The output voltage adjustment of the T31SN24005 is implemented through its TRIM pin, which operates by altering the voltage division ratio in the internal reference voltage circuit, typically achieved using resistive voltage dividers. This adjustment stabilizes the output voltage through an internal feedback loop. The accuracy of this adjustment depends on the value of the external resistor, which must be selected based on (1) to ensure the output voltage remains within the design range.

**Fig. 4:**
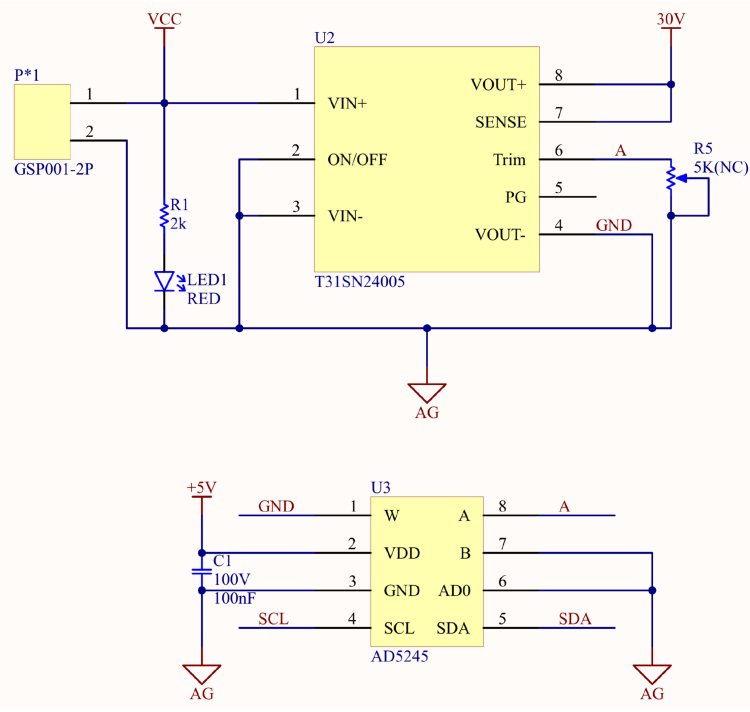
T31SN24005 power module and digital potentiometer.

The actual circuit configuration and wiring are illustrated in Fig. 4. A digital potentiometer controlled via I2C signals is used to precisely set the resistance values, enabling the T31SN24005 power module to execute different voltage control commands. This design allows the T31SN24005 power module to flexibly adapt to diverse application requirements, providing stable and precise output voltage control capabilities.

(1) $$R_{\text{trim}}=\left[\frac{21.9}{V_{o}-2.59}-0.511\right]\;\left(k\Omega \right)$$

### 2.4 Design of the voltage output and amplitude adjustment module

**Fig. 5:**
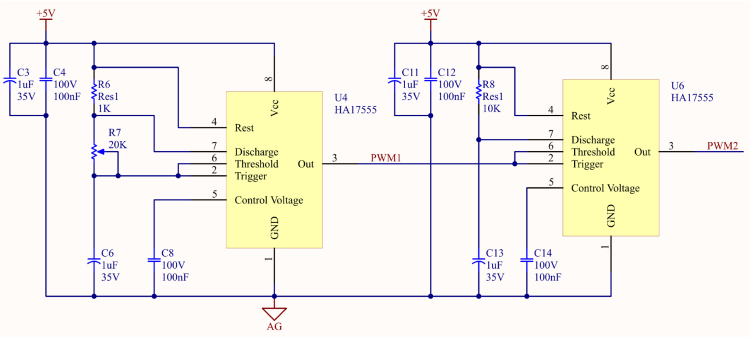
Master–Slave PWM signal generation circuit using dual NE555 timers.

The PWM signal generation circuit in this system as shown in Fig. 5 employs two NE555 timers configured in a master–slave architecture. The NE555 timer, a classic component in electronic circuit design, is widely favored for its high stability, low cost, and versatility. The master timer generates a stable clock signal, which is further processed by the slave timer to achieve precise control and flexible modulation of the PWM signal. This design is simple, easy to adjust, and effectively generates stable and adjustable PWM signals.

Through the master–slave architecture, this study fully exploits the high stability and multifunctional characteristics of the NE555 timer, resulting in a low-cost circuit that achieves both efficiency and stability. This architecture is particularly suitable for compact driver circuits and signal control systems, offering excellent design flexibility and practical value.

The master–slave PWM signal generation circuit is composed of two NE555 timers. The master timer (U4) is configured in an astable mode to generate a stable clock signal. According to Eq. (2), where $T_{h}$ represents the pulse high time, $T_{l}$ represents the pulse low time, and f represents the operating frequency, the output signal can be flexibly adjusted by varying the resistance values of $R_{6}$ and $R_{7}$ and the capacitance value of $C_{3}$. This clock signal serves as the trigger source for the slave timer (U6), which further processes the signal to generate precise and stable PWM signals, meeting the requirements of diverse applications.

(2) $$T_{h}=0.693\left(R_{6}+R_{7}\right)C_{3},$$

(3) $$T_{l}=0.693R_{7}C_{3},$$

(4) $$f=\frac{1.44}{\left(R_{6}+2R_{7}\right)C_{3}}.$$

The slave timer (U6) is configured in a monostable mode, where it remains in a low state while the master timer (U4) outputs a high state. When the master timer transitions to a low state, the slave timer is triggered and outputs a high state, generating complementary PWM signals. Additionally, the pulse width of the PWM signal can be adjusted using a potentiometer, enhancing control flexibility. The master timer generates a fixed-frequency square wave to trigger the slave timer, which produces a signal with a specific pulse width. The frequency and pulse width of the PWM signal can be controlled by adjusting the resistance of the external variable resistor $R_{7}$, thereby influencing the characteristics of the final AC square wave signal to meet precise application requirements.

In this design, the PWM signal plays a critical role by enabling the subsequent full-bridge architecture to convert DC power into AC square waves, which are used to drive Polymer Dispersed Liquid Crystal (PDLC) films. The AC square wave effectively regulates the alignment of liquid crystal molecules within the PDLC, allowing rapid switching between transparent and opaque states. This design not only features a simple structure but also demonstrates high energy conversion efficiency. Compared to traditional AC–AC drivers, it offers superior flexibility in adjustment and higher power density, making it suitable for diverse application scenarios.

### 2.5 UC3707 full-bridge driver circuit

To meet the requirements for driving current and AC driving voltage in the PDLC driver, this study employs the UC3707 as the core driver component. The UC3707 is a widely used high-current MOSFET or IGBT driver, and its circuit connection is shown in Fig. 5. This component integrates two half-bridge modules internally, which can be configured into a full-bridge architecture through external circuit design, demonstrating high integration and practicality.

**Fig. 6:**
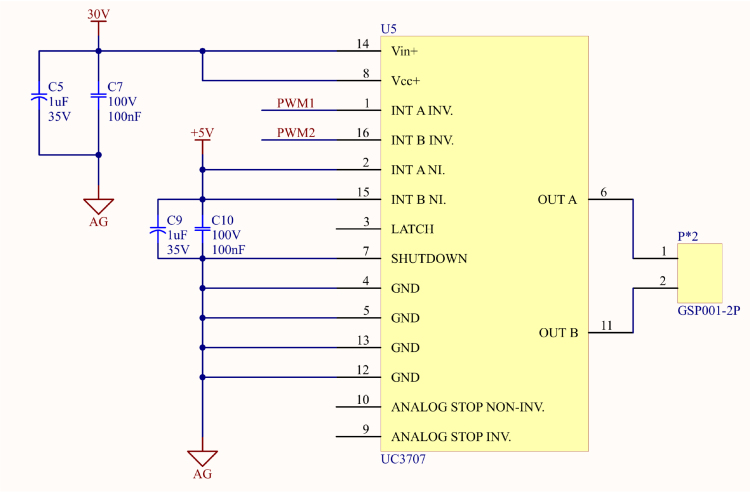
UC3707 full-bridge driver circuit.

The UC3707 features a high driving current of 1.5 A, a wide operating voltage range, and high operating frequencies. Additionally, it incorporates built-in over-temperature and over-current protection, significantly enhancing the reliability and safety of the circuit, making it an ideal choice for power electronics design. Furthermore, its dual-channel structure improves the flexibility and simplicity of circuit design, making it well-suited for a variety of application scenarios (see Fig. 6).

## 3 Design files summary

The design files include various resources related to the PDLC driver, offering comprehensive support for hardware and software development. These files are suitable for designing, manufacturing, and further development of the device. Among them, Drive_PCB V1.2 is a set of design files created using Altium Designer, consisting of schematics, layout, and project files. The schematics provide detailed descriptions of the circuit architecture and component connections, making them suitable for understanding the overall design logic or for making modifications. The layout files document the physical placement of components, routing, and design, and are intended for the actual manufacturing of the PCB. The project files contain complete design data, facilitating design management and rapid deployment.

The firmware section is supported by MiniDrive_Firmware, which contains firmware code for the ESP32 microcontroller, including .ino and .cpp files. The core function of this firmware is to establish I2C communication between the driver and the ESP32 microcontroller while also enabling users to perform secondary development based on specific requirements. This firmware plays a critical role in device control and data exchange, providing a robust foundation for efficient operation.

The developed driver also includes structural design resources with the PDLC_Power_top and PDLC_Power-_bottom files, which contain STL models for the upper and lower parts of the enclosure. These files are suitable for 3D printing and are crucial for ensuring mechanical stability and precise assembly of the device. The design of the enclosure not only enhances the structural integrity of the device but also effectively protects the internal electronic components.

All design files are published under an open-source license (CC BY 4.0), as shown in Table 1, and can be accessed and downloaded conveniently via the provided links. These files support academic research and engineering development, covering multiple aspects such as hardware design, software development, and mechanical fabrication. For viewing or editing these files, Altium Designer can be used for PCB files, Arduino IDE for editing and uploading firmware, and 3D printing machines for processing STL files. This comprehensive set of design files provides a complete solution for developing PDLC drivers and offers opportunities for further customization and secondary development.

**Table 1:** File information table.

Design filename	File type	Open source license	Location of the file
Drive_PCB V1.2	Schematics	CC BY 4.0	https://doi.org/10.5281/zenodo.14195829
Drive_PCB V1.2	Layout	CC BY 4.0	https://doi.org/10.5281/zenodo.14195829
Drive_PCB V1.2	Project	CC BY 4.0	https://doi.org/10.5281/zenodo.14195829
MiniDrive_Firmware	Firmware	CC BY 4.0	https://doi.org/10.5281/zenodo.14195829
PDLC_Power_top	CAD, 3D-printing	CC BY 4.0	https://doi.org/10.5281/zenodo.14195829
PDLC_Power_bottom	CAD, 3D-printing	CC BY 4.0	https://doi.org/10.5281/zenodo.14195829

## 4 Bill of materials summary

### 4.1 PCB parts

This subsection focuses on detailing the selection of all electronic components, including their specifications, quantities, purchasing sources, and prices as shown in Table 2.

**Table 2:** PCB components.

Designator	Comment	Quantity	Cost/unit (USD)	Total cost (USD)	Supplier	Supplier Part No.
C1, C3, C5, C7, C8, C11	E.CAP 1 μF 50 V DIP	6	0.15	0.9	Digi-Key	1189-1421-ND
C2, C4, C6, C9, C10, C12, C14	FILM CAP 0.1 μF 100 V DIP	7	0.28	1.96	Digi-Key	495-B32529C1104K008-ND
U2	T31SN24005	1	24.4	24.4	Digi-Key	941-T31SN24005NNFA-ND
U3	IC DGTL POT 5KOHM 256TAP SOT23-8	1	4.1	16.4	Digi-Key	AD5245BRJZ5-RL7DKR-ND
U4, U6	IC OSC SGLTIMER 100 kHz 8-SOIC	2	0.25	0.5	Digi-Key	296-6501-6-ND
U5	IC GATE DRVR LOW-SIDE 16DIP	1	9.7	9.7	Digi-Key	296-11243-5-ND
U1	DC–DC/LDO Module	1	3.67	3.67	MINMAX	M78AR05-1
LED1	LED RED CLEAR DIP	1	0.22	0.22	Digi-Key	C503B-RAN-CZ0C0AA1-ND
LED2	LED GREEN CLEAR DIP	1	0.22	0.22	Digi-Key	C503B-RAN-CZ0C0AA2CT-ND
P1	CONN HEADER R/A 2POS 2.54MM	1	0.43	0.43	Digi-Key	900-0022053021-ND
P*1, P*2	GSP001-5.08-2P	2	0.37	0.74	JIN HUA	2001300000767
R1	RES 2K OHM 5% 1 W AXIAL	1	0.34	0.34	Digi-Key	13-RSF100JB-73-2K-ND
R2, R3	RES 1.5K OHM 5% 1/2 W AXIAL	2	0.12	0.24	Digi-Key	CF12JT1K50TR-ND
R4	RES 330 OHM 5% 1/2 W AXIAL	1	0.12	0.12	Digi-Key	S330HTR-ND
R5	TRIMMER 5K OHM 0.5 W PC PIN TOP	1	1.97	1.97	Digi-Key	118-PV36W502C01B00-ND
R6	RES 1K OHM 5% 1/2 W AXIAL	1	0.12	0.12	Digi-Key	CF14JT1K00TR-ND
R7	TRIMMER 20K OHM 0.5 W PC PIN TOP	1	1.97	1.97	Digi-Key	118-PV36W203C01B00-ND
R8	RES 10K OHM 5% 1/2 W AXIAL	1	0.12	0.12	Digi-Key	CF14JT10K0TR-ND

### 4.2 Enclosure parts

This subsection provides detailed specifications and quantities of screws required for assembling the enclosure, along with purchasing sources and prices as shown in Table 3.

**Table 3:** Enclosure components.

Designator	Component	Number	Cost/unit (USD)	Total cost (USD)	Source	Material type
Screw1	M3*15 mm Stainless Steel Phillips Head Screws	4	0.108	0.432	McMaster-Carr 92000A124	Stainless steel
Screw2	M3*5 mm Stainless Steel Phillips Head Screws	4	0.0545	0.218	McMaster-Carr 92000A114	Stainless steel

## 5 Build instructions

1. Before operating the PDLC micro driver, ensure a DC power supply is used, and set the input voltage to 36V_DC_ (at this step, the DC power supply will not output voltage). Connect the positive and negative terminals of the power supply to the (P*1) terminal.
2. Connect the I2C pins (SDA, SCL) and GND of the driver to the corresponding pins of the controller.
3. Download and install the Arduino IDE.
4. Turn on the DC power supply.
5. Use the Arduino IDE to open the .ino program file from the MiniDrive Firmware folder.
6. Connect the controller to the computer via a USB cable. In the Arduino IDE, select the correct COM port under the Tools menu.

## 6 Operation instructions

1. Download and install the AD5245.h library.
2. Compile and upload the program.
3. Turn on the terminal interface to verify the driver has successfully connected.
4. Turn on the power supply and input 36 V_DC_ to the PDLC micro driver.
5. Ensure the terminal page is ready.
6. Send the commands 115200, to initialize the driver.
7. At the terminal, input any command and press ENTER to send.
8. Input values from 0-255 to adjust the AC output voltage amplitude from 5 V to 30 V.

## 7 Validation and characterization

### 7.1 Experimental validation

**Fig. 7:**
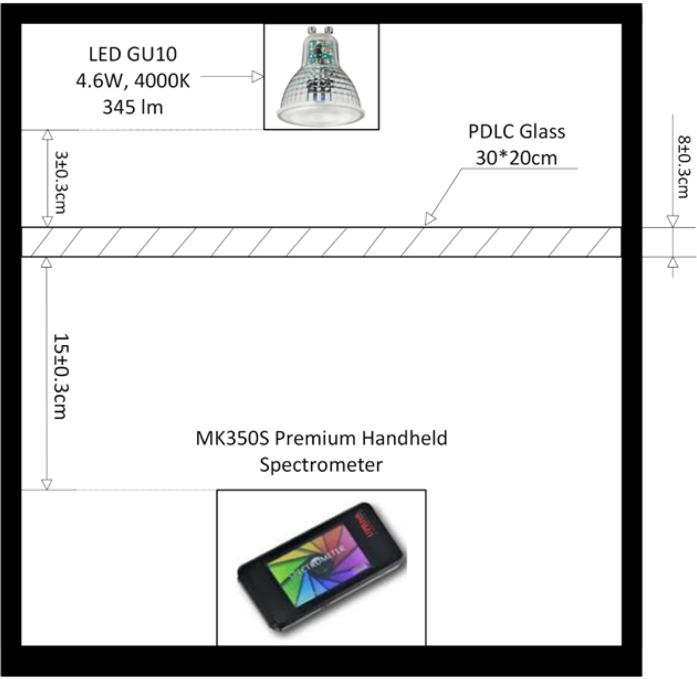
Illustration of the experimental setup for PDLC driver performance testing.

**Fig. 8:**
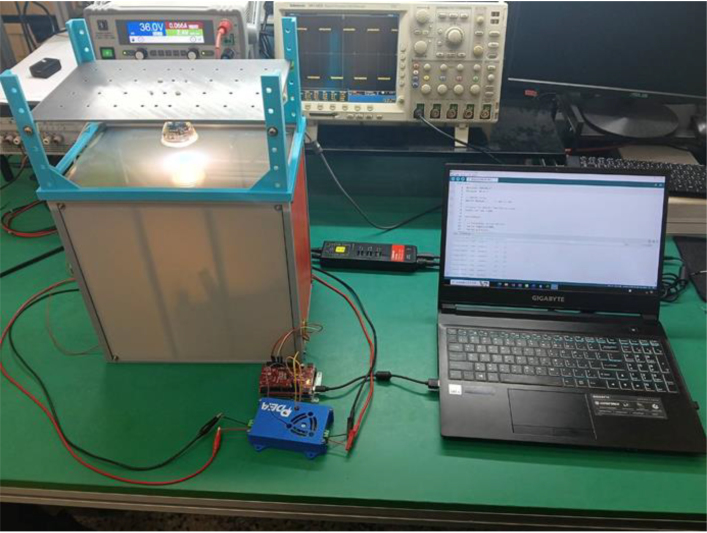
Experimental setup for PDLC driver control and measurement system.

To comprehensively verify the performance of the designed PDLC driver, a complete experimental setup was established, as shown in Fig. 7. The primary objective of this experiment is to evaluate the control effectiveness of the driver circuit, particularly its impact on the light transmittance of PDLC under varying driving conditions. To ensure accuracy and stability throughout the experimental process, a dedicated testing fixture was fabricated using 3D printing technology (as shown in Fig. 8). This fixture is used to securely mount the PDLC glass and the associated sensors, creating a stable and reproducible testing environment.

For optical measurements, the MK350S Premium spectrometer was employed to record spectral data during the light transmission process of the PDLC. This allowed for an in-depth analysis of the optical properties and spectral characteristics of the PDLC under different operating states. The light source used was the IKEA TRADFRI N LED GU10 bulb, which delivers 345 lumens of luminous flux, equivalent to the brightness of a traditional 50 W halogen bulb. It also features adjustable light color functionality, making it adaptable for various testing scenarios. This light source provides stable and uniform illumination, which is crucial for accurately measuring the transmittance of the PDLC glass.

During the experiment, the PDLC glass was first secured in the testing fixture, ensuring a precise and consistent distance between the light source, the PDLC glass, and the sensors to improve measurement reproducibility. Next, the driving circuit was used to adjust the driving voltage and frequency of the PDLC, and the MK350S Premium spectrometer was utilized to record data on the transmittance variation with voltage. With its high precision, the spectrometer provided detailed data on transmittance and spectral characteristics for analysis. Finally, multiple tests were conducted to ensure data consistency and reliability, thereby further validating the performance and stability of the designed PDLC driver circuit.

**Fig. 9:**
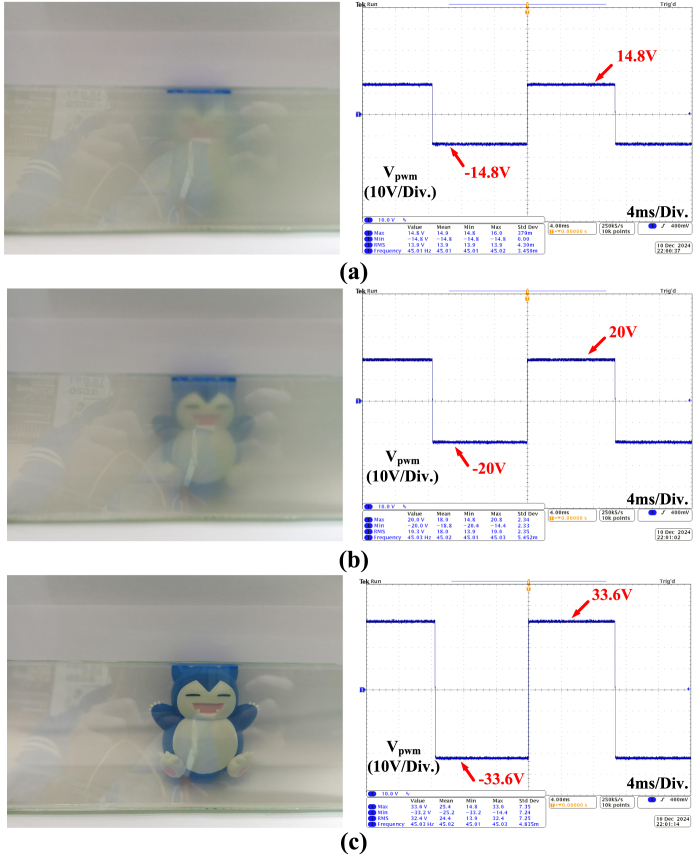
(a) Measured image of PDLC glass at 40% transmittance with corresponding output voltage waveform (b) Measured image of PDLC glass at 50% transmittance with corresponding output voltage waveform (c) Measured image of PDLC glass at 65% transmittance with corresponding output voltage waveform.

This study evaluates the impact of various driving voltages on the light transmittance performance of PDLC and verifies whether it meets the requirements for dimming applications. The driving voltage range was set between 5 V and 30 V to observe the response characteristics of PDLC material under different voltage conditions. To ensure data reliability and accuracy, each measurement was repeated three times, and the average value was taken to minimize measurement errors. The experimental results, as shown in Fig. 9, indicate that as the driving voltage increases, the light transmittance of PDLC gradually rises and saturates at a specific voltage range. This result aligns with the data provided in the PDLC material datasheet, confirming that the designed driver circuit can stably and effectively control the light transmittance of PDLC.

**Fig. 10:**
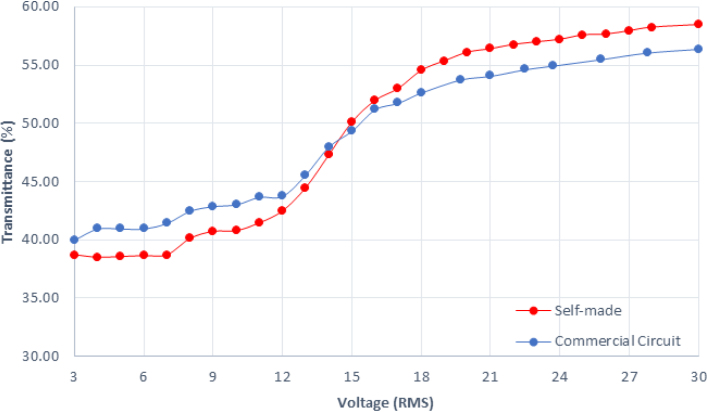
Comparison of PDLC transmittance under varying driving voltages between the self-made driver and a commercial circuit.

Furthermore, a comparison of the transmittance control performance between the custom-designed driver circuit and commercially available power supplies was conducted, as illustrated in Fig. 10. The results demonstrate that the custom circuit achieves comparable performance to commercial products under low power consumption conditions. This highlights the advantages of the proposed driver circuit in terms of energy efficiency and performance control, further proving the feasibility and application potential of this design.

### 7.2 Conclusion

The proposed adjustable micro PDLC driver embodies a forward-looking approach to reducing energy consumption and optimizing resource efficiency in modern smart building systems. By integrating IoT capabilities for remote control, the driver enables precise light transmittance regulation across a voltage range of 5 V to 30 V, significantly enhancing operational flexibility . Its compact, modular design not only minimizes material usage but also supports multi-zone applications such as smart windows and privacy glass, contributing to energy savings in diverse scenarios. Experimental results validate its stability and effectiveness, demonstrating comparable performance to commercial drivers while operating at reduced power levels. This innovation reflects a commitment to developing scalable, energy-conscious technologies that align with global efforts to create more sustainable solutions for future infrastructure and urban systems.

## CRediT authorship contribution statement

**Kun-Che Ho:** Writing – review & editing, Supervision, Resources, Methodology, Conceptualization. **Rui-Feng Xu:** Writing – original draft, Software, Project administration, Data curation. **Cheng-Xun Wu:** Validation, Methodology, Data curation, Conceptualization. **Jia-Zheng Liao:** Validation, Resources.

## Declaration of competing interest

The authors declare that they have no known competing financial interests or personal relationships that could have appeared to influence the work reported in this paper.
